# Analysis of Function Role and Long Noncoding RNA Expression in Chronic Heart Failure Rats Treated with Hui Yang Jiu Ji Decoction

**DOI:** 10.1155/2023/7438567

**Published:** 2023-01-17

**Authors:** Huan Zhang, Li Zhang, Kejing Yin, Miao Zhang, Yanjiao Hao, Hongwei Zhou, Huifeng Ren

**Affiliations:** The Second Affiliated Hospital of Shaanxi University of Chinese Medicine, 5 Weiyang Xi Road, Qindu District, Xianyang, Shaanxi 712000, China

## Abstract

Hui Yang Jiu Ji (HYJJ) decoction has been applied as a prescription of traditional Chinese medicine for the treatment of chronic heart failure (CHF). However, its comprehensive molecular mechanism remains unclear now. Our study aimed to explore the possible function and lncRNA-miRNA regulation networks of HYJJ on CHF induced by doxorubicin (DOX) in rats. Our study showed that HYJJ could recover cardiac function and alleviate myocardial injury of DOX-induced CHF. Besides, HYJJ had an effect on restraining myocardial apoptosis in CHF rats. Moreover, RNA-sequencing and bioinformatics analysis indicated that among a total of 548 significantly up- and down-regulated differentially expressed (DE) long noncoding RNA (lncRNA), 511 up- and down-regulated DE miRNAs were identified. Cushing's syndrome and Adrenergic signaling in cardiomyocytes were common pathways between DE-lncRNAs-enriched pathways and DE-miRNAs-enriched pathways. Finally, we observed a new pathway-MSTRG.598.1/Lilrb2 pathway with the HYJJ treatment; however, it needs further studies. In conclusion, this study provided evidence that HYJJ may be a suitable medicine for treating CHF. Moreover, several pivotal miRNAs may serve important roles in these processes by regulating some key miRNAs or pathways in CHF.

## 1. Introduction

Chronic heart failure (CHF) was defined as a group of clinical syndromes caused by ventricular filling or impaired ejection ability when some cardiac diseases develop to the terminal stage [1]. Owing to the characteristics of high morbidity, disability, and mortality [2], CHF is still the leading cause of death and will increase the medical care burden in the next few decades [3, 4]. Doxorubicin (DOX) was restricted partly on account of its cardiotoxicity and high incidence of severe heart failure [5]. DOX-induced cardiotoxicity was irreversible, consequently progressing to the development of CHF, dilated cardiomyopathy, and left ventricular dysfunction [6]. Therefore, the identification of more effective therapeutic approaches to CHF was urgently needed.

Hui Yang Jiu Ji decoction (HYJJ) was a traditional decoction often used for patients with acute gastroenteritis, excessive vomiting, diarrhea, shock, myocardial infarction, heart failure, and some acute symptoms. For example, Jin et al. [7] reported that HYJJ could increase the left ventricular ejection fraction (LVEF) of patients with chronic heart failure. Zeng et al. [8] reported that HYJJ had a good clinical efficacy on cardiogenic shock complicating acute myocardial infarction. However, the underlying mechanism of HYJJ remained unclear.

Transcriptome sequencing utilizes high-throughput sequencing technology to reflect the expression levels of miRNA and noncoding RNA [9]. Transcriptomic was an omics technology that studied the types and structures of all gene transcripts in a specific time and space from an overall level. It was a method that could reveal the situation of gene transcription, the regulation rules of genes, and the molecular mechanism in the occurrence and development of diseases [10]. Besides, transcriptomics was an important part of functional genomics and has been widely used in many fields such as life sciences and medicine [11]. In the field of traditional Chinese medicine (TCM), transcriptomic was often used to explore the functional genes of TCM and study the metabolic pathways of effective ingredients [12, 13]. Therefore, the function of HYJJ on CHF and its molecular mechanism was explored in this study based on transcriptome sequencing.

In the present study, we investigated the cardioprotective effect of HYJJ in DOX-induced CHF *in vivo*. In addition, transcriptome sequencing technology was used to analyze the lncRNA/miRNAs expressions of CHF model rats after HYJJ treatment in order to find the possible molecular mechanism and key targets of HYJJ intervention in CHF.

## 2. Materials and Methods

### 2.1. Drugs and Reagents

Hui Yang Jiu Ji decoction (HYJJ) was provided by the Second Affiliated Hospital of Shaanxi University of Traditional Chinese Medicine. Digoxin was purchased from Shanghai Shangyao Xinyi Pharmaceutical Factory Co., Ltd (Shanghai, China). HYJJ and digoxin were administered at 7.25 g/kg/d and 0.027 mg/kg/d by intragastric administration, respectively. DOX hydrochloride was obtained from Shanxi Pude Pharmaceutical Co., Ltd (Shanxi, China) and configured as a test solution with a concentration of 0.8 mg/ml by 0.9% saline.

### 2.2. Rats Model of CHF and Treatments

Male Sprague–Dawley (SD) rats, weighing 200 ± 20 g, were obtained from Shanghai Slack Laboratory Animal Co., Ltd. (Shanghai, China) and housed under a 12 h–12 h dark/light cycle at 21 ± 2°C and 30–70% relative humidity, with freely available rodent chow and water.

Rats in the CHF groups were intraperitoneally injected with DOX hydrochloride at a concentration of 1.5 mg/kg (twice a week for four weeks). Correspondingly, the control group was intraperitoneally injected with saline. After the successful construction of the CHF model, rats were divided into the following three groups: CHF, HYJJ (7.25 g/kg), and digoxin (0.027 mg/kg). HYJJ and digoxin groups were administered intragastrically with corresponding drugs once a day for two consecutive weeks. The rats in the control and CHF groups received an equal volume of normal saline.

### 2.3. Measurement of Cardiac Function

Rats were anesthetized using 1% sodium pentobarbital, then the left ventricular internal diameter at end-diastole (LVIDd), left ventricular internal diameter at end-systole (LVIDs), ejection fraction (EF), and fractional shortening (FS) were measured by transthoracic echocardiography using a Vivid-i echocardiography machine (General Electric Company) equipped with an 11.5 MHz transducer.

### 2.4. Terminal-DeoxynucleoitidylTransferase-Mediated Nick End Labeling (TUNEL) Assay

The myocardial tissues were collected and embedded in paraffin and sliced into sections. The tissues were then subjected to the One Step TUNEL Apoptosis Assay Kit on the basis of the manufacturer's protocol (Beyotime, China). TUNEL-positive cells were imaged under a fluorescence microscope (Nikon, Tokyo, Japan).

### 2.5. Hematoxylin-Eosin (HE) Staining

The myocardial tissues were fixed in 4% paraformaldehyde, embedded in paraffin, sliced into 5 *μ*m sections, deparaffinized, and rehydrated as previously described. After rehydration, the sections were stained with HE Staining Kit followed by the manufacturer's instructions (Invitrogen, USA). The stained sections were then viewed under the ordinary optic microscope (Nikon, Tokyo, Japan).

### 2.6. Immunofluorescence Staining

The paraffin sections of myocardial tissues were dewaxing, alcohol dehydration, and antigen retrieval. Then, the sections were washed three times for five minutes each time by PBST and blocked by 2% BSA. Sections were then treated with primary antibody cleaved caspase-3 (c-caspase 3, Cell signaling, USA) at 4°C overnight. After six times washing, the sections were incubated with fluorescein isocyanate (FITC)-conjugated goat anti-mouse (KPL, USA) as a secondary antibody at 37°C for 30 min. Finally, images were acquired using a fluorescent microscope (Nikon, Tokyo, Japan) at 100x magnification. Moreover, the expression level was quantified by mean fluorescence intensity (MFI).

### 2.7. Enzyme-Linked Immunosorbent Assay (ELISA)

Serum from rats in each group was collected, and the levels of cardiac troponin I (cTNI) and N-terminalpro-brain natriuretic peptide (NT-proBNP) were determined by ELISA based on the manufacturer's instructions. The ELISA kits were both purchased from Shanghai Kanglang Biological Technology Co., Ltd (Shanghai, China). The OD value of each well was immediately read at 450 nm.

### 2.8. Western Blot Assay

To confirm the expression level of c-caspase 3 in myocardial tissues, myocardial tissues were lysed with RIPA buffer. Lysates were separated on 10% SDS-polyacrylamide gel and proteins were transferred to polyvinylidene difluoride (PVDF) membranes. Then, the membranes were blocked with 5% nonfat milk for 1 h at room temperature, and membranes were incubated with the corresponding primary antibody at 4°C overnight following incubation with horseradish peroxidase-conjugated secondary antibody at room temperature for 1 h. Protein expression was detected with high sensitivity ECL chemiluminescence detection kit (Vazyme, China). The bands' intensity was expressed as fold change by normalizing the data to the values of *β*-actin using ImageJ software.

### 2.9. Transcriptome Sequencing

The sequencing work was performed by Shanghai Paisennuo Biotechnology Co. LTD. In brief, total RNA was extracted from the myocardial tissue and removed rRNA. The extracted RNA was interrupted to a 300 bp fragment by ion interruption and constructed the library through reverse transcription. Then, the library was sequenced using the next-generation sequencing (NGS) and Illumina HiSeq Sequencing platform. Clean data obtained by filtering raw data were used for the identification of lncRNA and mRNA. Finally, Gene Ontology (GO) analysis and Kyoto Encyclopaedia of Genes and Genomes (KEGG) analysis were used for the prediction of action pathways. The analysis data has been uploaded to GitHub (https://github.com/Zhanghuanhaoxuexi/Analysis-of-function-role-and-long-non-coding-RNA-expression-in-chronic-heart-failure-rats-treated-w, No: dc211eb).

### 2.10. Statistical Analysis

Statistical analysis was conducted by SPSS22.0 and GraphPad Prism 9 software. Data were shown as means ± standard deviation (SD). One-way analysis of variance (ANOVA) or two-way ANOVA was used to compare the difference among multiple groups followed by post hoc tests. *p* < 0.05 was thought to be statistically significant.

## 3. Results

### 3.1. Hui Yang Jiu Ji Decoction Alleviated Myocardial Injury of CHF Rats

**Figure 1:**
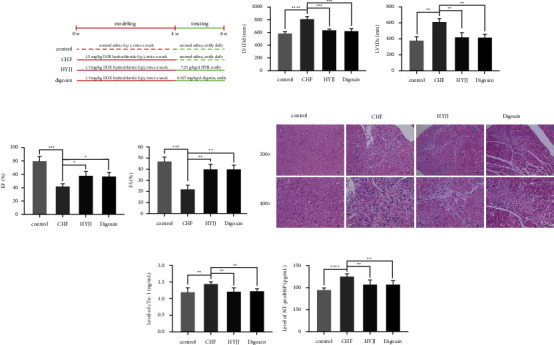
Hui Yang Jiu Ji decoction alleviated myocardial injury of CHF rats: (a) The diagram of modeling and grouping in this experiment. (b) Echocardiographic LVIDd and LVIDs values were detected in each group following CHF. (c) Percentages of EF and FS were calculated in each group after CHF. (d) HE staining of myocardial tissues in each group at a magnification of 200x and 400x. Scale bar = 10 *μ*m. (e) Expression levels of cTNI and NT-proBNP in each group were determined by ELISA assay. Bars represented the mean ± S.D. from at least three independent experiments. ^*∗*^*p* < 0.05, ^*∗∗*^*p* < 0.01, ^*∗∗∗*^*p* < 0.001, and ^*∗∗∗∗*^*p* < 0.0001.

The diagram of modeling and grouping in this experiment is shown in Figure 1(a). For exploring the cardioprotection function of HYJJ, HE staining and ELISA assay were conducted. The LVIDd and LVIDs values or EF and FS percentages were markedly increased or decreased in the CHF group, suggesting the damage to cardiac function and initiation of CHF; however, HYJJ and digoxin treatment prominently reverse the trend above (Figures 1(b) and 1(c)) (Table S1). Next, compared with the control group, the CHF group had more myocardial fiber necrosis, nuclear pyknosis, and disintegration. Besides, a small amount of inflammatory cell infiltration was observed in the necrotic area and stroma. It was mainly composed of lymphocytes with round and deep stained mononuclear cells, and fibrous tissue proliferation was also seen, including the proliferation of fibroblasts with long oval nuclei and fibroblasts with long spindle-shaped nuclei. However, HYJJ and digoxin treatment could alleviate the pathological injury of CHF (Figure 1(d)) (Table S1). Similarly, the expression levels of myocardial injury markers, including cTNI and NT-proBNP were both significantly increased in the CHF group, but the obviously decreased trend of cTNI and NT-proBNP was observed after HYJJ and digoxin treatment (Figure 1(e)) (Table S1). These data suggested that HYJJ might play a protective role in CHF.

### 3.2. Hui Yang Jiu Ji Decoction Inhibited Myocardial Apoptosis of CHF Rats

**Figure 2:**
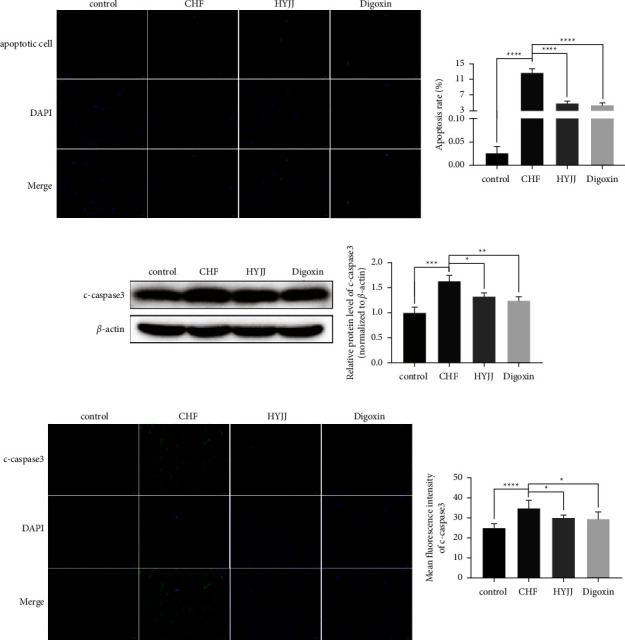
Hui Yang Jiu Ji decoction inhibited myocardial apoptosis of CHF rats: (a) The TUNEL-negative normal cells with blue nuclear fluorescence (DAPI) and TUNEL-positive cells with green nuclear fluorescence at a magnification of 400x. Scale bar = 20 *μ*m. The results of the TUNEL assay were quantified by apoptosis rate in each group. (b) The WB band of c-caspase 3 was shown, and the relative expression level was normalized to *β*-actin. (c) The expression of c-caspase 3 was dyed green, and the cell nucleus was stained blue at a magnification of 100x. Scale bar = 100 *μ*m. The level of c-caspase 3 was quantified by MFI. Bars represented the mean ± S.D. from at least three independent experiments. ^*∗*^*p* < 0.05, ^*∗∗*^*p* < 0.01, ^*∗∗∗*^*p* < 0.001, and ^*∗∗∗∗*^*p* < 0.0001.

To further explore the function of HYJJ in CHF rats, TUNEL assay and WB were performed. The data showed that the obviously enhanced apoptosis rate was observed in the CHF group, while HYJJ and digoxin treatment could prominently downregulate the apoptosis rate (Figure 2(a)). Additionally, the relative expression level and MFI of c-caspase 3 were both significantly increased in the CHF group, but this phenomenon was markedly reverted with elevated c-caspase 3 expressions both in HYJJ and digoxin group (Figures 2(b) and 2(c)) (Table S2). These results indicated that the occurrence of apoptosis was found in CHF, and HYJJ could relieve the apoptosis phenomenon caused by CHF.

### 3.3. Effect of Hui Yang Jiu Ji Decoction on lncRNA Expression Profiles

**Figure 3:**
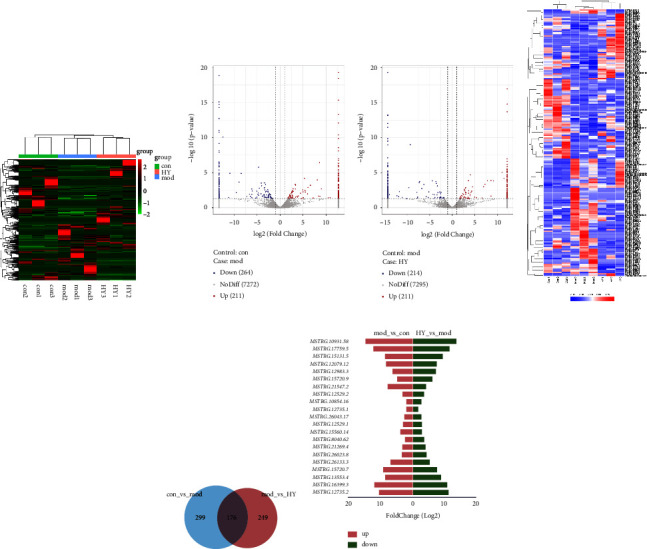
Analysis of lncRNA DE genes in the mod vs. con and HY vs. mod comparisons: (a) Clustering heat map of total lncRNA genes in three groups. (b) Volcano plot of lncRNA DE expression analysis. (c) Clustering heat map of DE lncRNAs genes. (d) Venn diagram showing lncRNA DE genes. (e) Top 10 upregulated lncRNA DE genes and the top 10 downregulated lncRNA DE genes in HY vs. mod comparison. Con: control group, mod: CHF group, and HY: HYJJ group.

In this part, we analyzed the effect of HYJJ decoction on the expression of lncRNA in CHF rats through transcriptome. After normalizing the abundance of lncRNA genes, the distribution of total lncRNA genes was provided in Figure 3(a). As shown in Figure 3(b), the upregulation of 211 lncRNA genes and downregulation of 264 lncRNA genes were revealed in the con vs. mod comparison, and the upregulation of 211 lncRNA genes and downregulation of 214 lncRNA genes in the HY vs. mod comparison. Then, significantly differentially expressed (DE) genes were identified on the basis of |log2 (foldchange)| ≥ 1 and FDR ≤ 0.05. As shown in Figure 3(c), the expression patterns of lncRNAs were mostly consistent within each group; however, they differed significantly between the con, mod, and HY groups. Moreover, the number of lncRNAs DE genes specific to the mod vs. con and HY vs. mod comparisons were 299 and 249, respectively, and 176 DE genes were common to both two comparison groups (Figure 3(d)). Moreover, the top 10 upregulated lncRNA DE genes and the top 10 downregulated lncRNA DE genes in HY vs. mod comparison were shown in Figure 3(e). The top 3 upregulated lncRNA DE genes were MSTRG.12735.2, MSTRG.16399.3, and MSTRG.13553.4, and the top 3 downregulated lncRNA DE genes were MSTRG.12735.1, MSTRG.10854.16, and MSTRG.12529.2.

**Figure 4:**
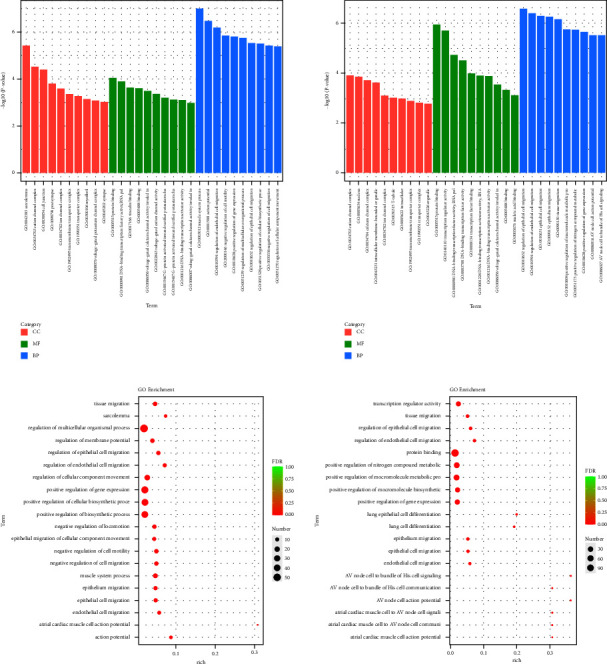
Gene ontology (GO) analysis of lncRNA DE genes: (a) GO enrichment analysis of lncRNA DE genes. (b) GO rich-factor analysis of lncRNA DE genes. Con: control group, mod: CHF group, and HY: HYJJ group.

The Gene Ontology (GO) functional enrichment results showed that HYJJ affected the biological processes (BP), cellular components (CC), and molecular functions (MF) of CHF rats' hearts (Figure 4(a)). Compared with the con group, the mod group had DE lncRNA genes primarily in regulation of the multicellular organismal process, positive regulation of gene expression, positive regulation of the cellular biosynthetic process, and positive regulation of the biosynthetic process. Compared with the mod group, the HY group had lncRNA DE genes primarily enriched in protein binding, positive regulation of nitrogen compound metabolic, positive regulation of macromolecule metabolic process, positive regulation of macromolecule biosynthetic, and positive regulation of gene expression (Figure 4(b)).

**Figure 5:**
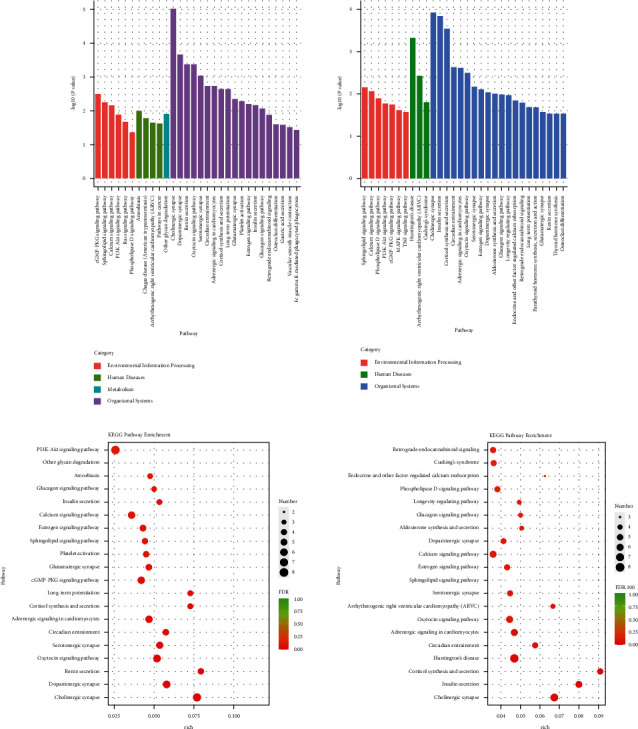
Kyoto Encyclopaedia of genes and genomes (KEGG) analysis of lncRNA DE genes: (a) KEGG pathway enrichment analysis of lncRNA DE genes. (b) KEGG pathway rich-factor analysis of lncRNA DE genes. Con: control group, mod: CHF group, and HY: HYJJ group.

The specific signaling pathways enriched with lncRNA DE genes were identified using tools at the Kyoto Encyclopaedia of genes and genomes (KEGG) database. As shown in Figure 5(a), the top 30 pathways the lncRNA DE genes significantly enriched were shown. As shown in Figure 5(b), the number of the lncRNA DE genes enriched in the differential pathways was shown. Compared with the con group, the lncRNA DE genes in the mod group were mainly enriched in the PI3K-Akt signaling pathway. Moreover, the differential pathways of the lncRNA DE genes mainly enriched in the HY group compared with the mod group were as follows: retrograde endocannabinoid signaling, Cushing's syndrome, endocrine, and other factor-regulated calcium reabsorption, phospholipase D signaling pathway, longevity regulating pathway, glucagon signaling pathway, dopaminergic synapse, calcium signaling pathway, estrogen signaling pathway, sphingolipid signaling pathway, serotonergic synapse, arrhythmogenic right ventricular cardiomyopathy (ARVC), oxytocin signaling pathway, adrenergic signaling in cardiomyocytes, circadian entrainment, Huntington's disease, cortisol synthesis and secretion, insulin resistance, and cholinergic synapse.

### 3.4. Effect of Hui Yang Jiu Ji Decoction on miRNA Expression Profiles

**Figure 6:**
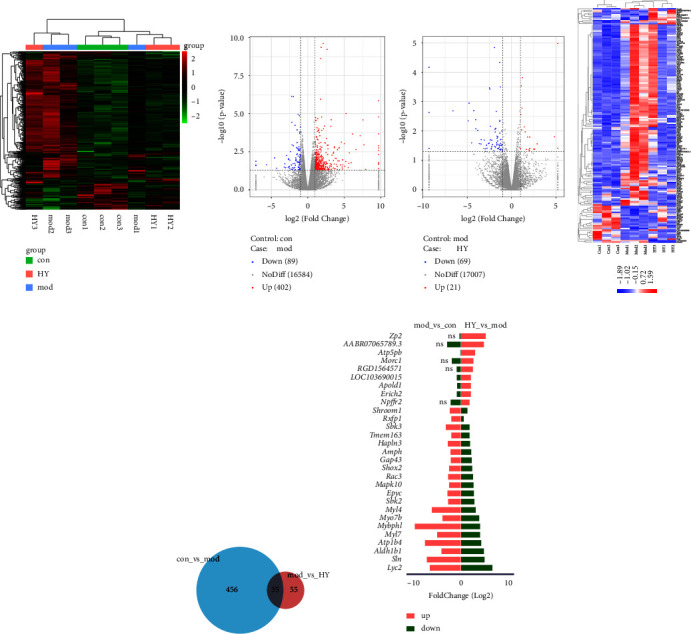
Analysis of miRNA DE genes in the mod vs. con and HY vs. mod comparisons: (a) Clustering heat map of total miRNA genes in three groups. (b) Volcano plot of miRNA DE expression analysis. (c) Clustering heat map of miRNAs DE genes. (d) Venn diagram showing common miRNA DE genes. (e) Top 20 upregulated miRNA DE genes and the top 20 downregulated miRNA DE genes (|log2 (fold change)| ≥ 2) in the HY vs. mod comparison. Con: control group, mod: CHF group, and HY: HYJJ group.

Next, we explored the effect of HYJJ decoction on miRNA expression profiles. The distribution of total miRNA genes is provided in Figure 6(a). As shown in Figure 6(b), there was upregulation of 402 miRNA DE genes and downregulation of 89 miRNA DE genes in the mod vs. con comparison, and upregulation of 21 miRNA DE genes and downregulation of 69 miRNA DE genes in the HY vs. mod comparison. As shown in Figure 6(c), there was a difference in miRNA DE gene expression patterns between the con, mod, and HY groups. The number of DE genes specific to the mod vs. con and HY vs. mod comparisons was 456 and 55, respectively, and 35 DE genes were common to both two comparison groups (Figure 6(d)). Moreover, the top 20 upregulated and the top 20 downregulated miRNA DE genes (|log2 (foldchange)| ≥ 2) in HY vs. mod comparison were shown in Figure 6(e), the top 3 upregulated miRNA DE genes were Zp2, AABRO7065789.3, and Atp5pb, and the top 3 downregulated miRNA DE genes were Lyc2, Sln, and Aldh1b1.

**Figure 7:**
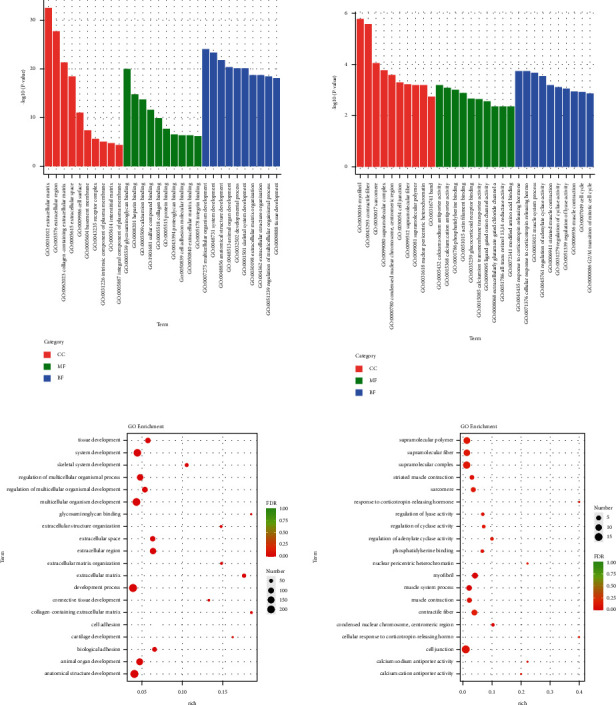
Gene ontology (GO) analysis of miRNA DE genes: (a) GO enrichment analysis of miRNA DE genes. (b) GO rich-factor analysis of miRNA DE genes. Con: control group, mod: CHF group, and HY: HYJJ group.

The gene ontology (GO) functional enrichment results showed that HYJJ affected the biological processes (BP), cellular components (CC), and molecular functions (MF) of miRNA in CHF rats' hearts (Figure 7(a)). Compared with the con group, the mod group had miRNA DE genes primarily in anatomical structure development, developmental process, multicellular organism development, and system development. Compared with the mod group, the HY group had miRNA DE genes primarily enriched in cell junction, supramolecular polymer, supramolecular fiber, and supramolecular complex (Figure 7(b)).

**Figure 8:**
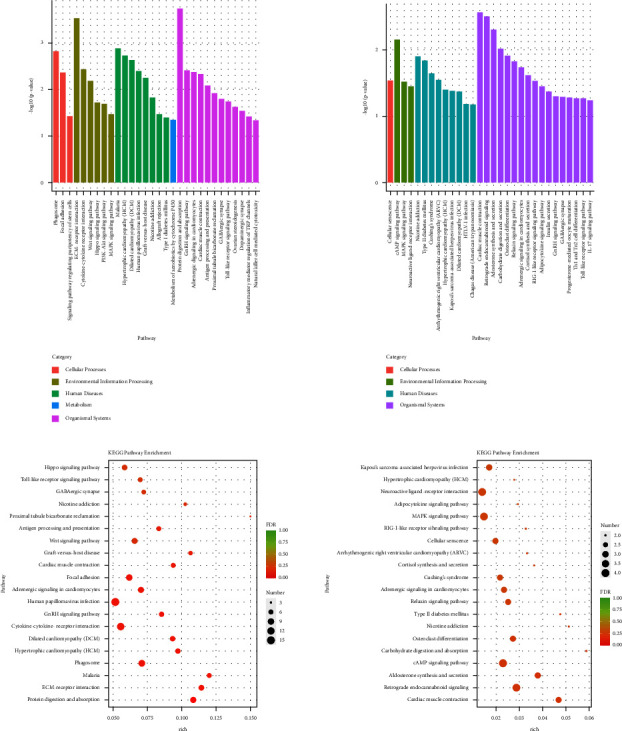
Kyoto Encyclopaedia of genes and genomes (KEGG) analysis of miRNA DE genes. (a) KEGG pathway enrichment analysis of miRNA DE genes. (b) KEGG pathway rich-factor analysis of miRNA DE genes. Con: control group, mod: CHF group, and HY: HYJJ group.

The specific signaling pathways enriched with miRNA DE genes were also identified using the KEGG database. As shown in Figure 8(a), the top 30 pathways of the miRNA DE genes significantly enriched were showing, and it found that hypertrophic cardiomyopathy (HCM) and dilated cardiomyopathy (DCM) both were the pathways significantly enriched with lncRNA DE genes in the two comparisons between the mod group and the con group and between the HY group and the mod group. As shown in Figure 8(b), the number of the miRNA DE genes enriched in the differential pathways was shown. In the con group compared with the mod group, HCM, DCM, and cardiac muscle contraction were the differential pathways of the miRNA DE genes mainly enriched. In the HY group compared with the mod group, the differential pathways of the miRNA DE genes mainly enriched were hypertrophic cardiomyopathy (HCM), neuroactive ligand-receptor interaction, adipocytokine signaling pathway, MAPK signaling pathway, RIG-I-like receptor signaling pathway, cellular senescence, arrhythmogenic right ventricular cardiomyopathy (ARVC), cortisol synthesis and secretion, Cushing's syndrome, adrenergic signaling in cardiomyocytes, relaxin signaling pathway, type II diabetes mellitus, nicotine addiction, osteoclast differentiation, carbohydrate digestion and absorption, cAMP signaling pathway, aldosterone synthesis and secretion, retrograde endocannabinoid signaling, and cardiac muscle contraction. Interestingly, HCM and cardiac muscle contraction both were the pathways lncRNA DE genes mainly enriched in the two comparisons; therefore, HCM and cardiac muscle contraction might be a key role in HYJJ treatment.

**Figure 9:**
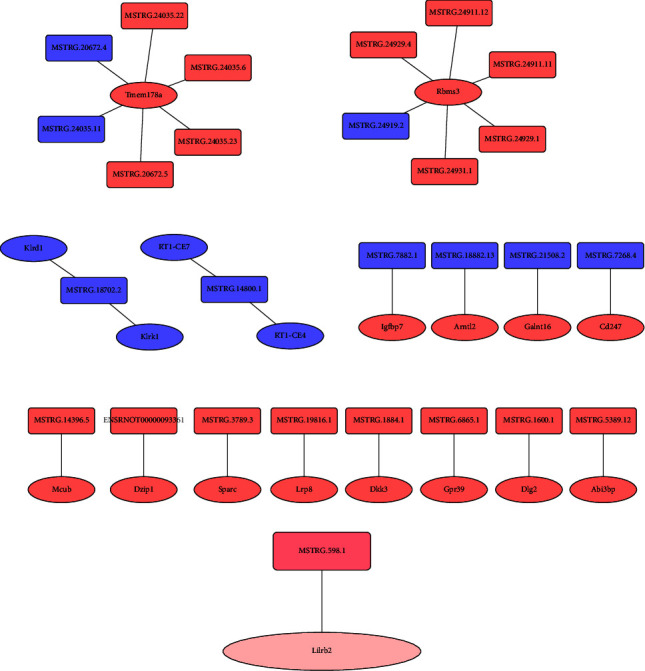
The potential interaction of miRNA and lncRNA. (a) The potential interaction of miRNA and lncRNA in the mod vs. con. Rectangles represent lncRNA, ellipses represent miRNA. Orange-red: upregulation, dark-blue: downregulation. (b) The potential interaction of miRNA and lncRNA in the HY vs. mod. Rectangles represent lncRNA, ellipses represent miRNA. Dark pink: upregulation, dark pink: downregulation. Con: control group, mod: CHF group, and HY: HYJJ group.

Finally, the potential interactions of miRNA and lncRNA were predicted. Coding-noncoding gene coexpression networks (CNC networks) were drawn based on the correlation analysis between miRNAs and miRNAs. There were many lncRNA-miRNA pairs that act on each other in both cis- and trans-fashion in the mod vs. con comparison (Figure 9(a)); however, only one lncRNA-miRNA pair MSTRG.598.1-Lilrb2 that acts on transfashion was observed in the HY vs. mod comparison (Figure 9(b)).

## 4. Discussion

This study has shown that the HYJJ could inhibit myocardial apoptosis and alleviate myocardial injury in DOX-induced CHF rats. Then transcriptome sequencing technology was used to analyze the lncRNA/miRNAs expressions of CHF model rats after HYJJ treatment. Through DE lncRNAs and DE miRNA analyses, we found HYJJ had an impact on the biological processes (BP), cellular components (CC), and molecular functions (MF) of CHF rats' hearts, and HYJJ improved DOX-induced CHF via multiple signaling pathways.

The apoptosis of cardiomyocytes is known to be an important mechanism of myocardial injury. Many studies showed that myocardial apoptosis was involved in the pathogenesis of acute myocardial infarction [14], ischemia/reperfusion injury in cardiomyocytes [15], and a series of cardiovascular ailments [16]. In our study, CHF increased myocardial apoptosis rate and c-caspase 3 expression, whereas HYJJ treatment reversed these effects and alleviated myocardial injury. This finding demonstrated that HYJJ could treat CHF effectively by reducing myocardial apoptosis.

As previously reported, transcriptional profiling has been utilized extensively in human heart failure to explore the new pathways involved in the complex disease [17], identify novel biomarkers for better diagnostic and prognostic accuracy [18], and evaluate the treatment responses of different medications or implanted devices [19–21]. A considerable amount of research has indicated that lncRNAs play important roles in many diseases [22–25]. Ischemic cardiomyopathy (ICM) and dilated cardiomyopathy (DCM) are two major problems that lead to heart failure ultimately. Recently, some studies demonstrated that lncRNA dysregulation in hearts suffered from ICM and DCM [26–28], indicating lncRNAs are closely involved in the cardiac pathophysiological processes. Our study revealed 475 DE lncRNAs in CHF rats compared with the control rats and a further 435 DE lncRNAs in HYJJ-treated rats compared with CHF rats, supporting the notion that lncRNAs are closely involved in the development of CHF and revealing the regulation of HYJJ to lncRNAs in CHF. In addition, lncRNAs were potentially new and crucial regulators in gene expression, transcription, post-transcription, and epigenetics levels [29]; among these, modulation of miRNA represents one of the most important actions of lncRNA in diseases. Our study showed 491 miRNA DE genes in CHF rats compared with the control rats and 90 miRNA DE genes in HYJJ-treated rats compared with CHF rats. In addition, the GO analysis revealed that DE lncRNAs were mainly enriched in protein binding, positive regulation of nitrogen compound metabolic, positive regulation of macromolecule metabolic process, positive regulation of macromolecule biosynthetic, and positive regulation of gene expression, and DE miRNA were mainly enriched in cell junction, supramolecular polymer, supramolecular fiber, supramolecular complex. These data provide direct evidence that HYJJ has an impact on the biological processes (BP), cellular components (CC), and molecular functions (MF) of DOX-induced CHF rats' hearts.

KEGG results showed that the lncRNA DE genes were mainly enriched in the PI3K-Akt signaling pathway in the mod vs. con comparison. In cardiac hypertrophy, the PI3K-Akt signaling pathway is activated by many types of cellular stimuli or toxic insults to regulate fundamental cellular processes including protein synthesis, proliferation, and survival, via its downstream signals, such as AKT, GSK3*β*, mTOR, P70S6K, and eIF-4E [30–32]. Moreover, the PI3K-Akt signaling pathway is involved in regulating cardiomyocyte apoptosis [33, 34], which plays an important role in the development of heart failure. Our data showed the PI3K-Akt signaling pathway might contribute to cardiomyocyte apoptosis to promote CHF, in line with previous findings [33, 34], once again indicating the importance of the PI3K-Akt signaling pathway in CHF development.

Further KEGG results showed that there were 13 pathways significantly enriched with lncRNA DE genes and 18 pathways significantly enriched with miRNA DE genes in the HY group compared with the mod group. Among them, Cushing's syndrome and Adrenergic signaling in cardiomyocytes were common pathways between lncRNAs-enriched pathways and miRNAs-enriched pathways.

Glucocorticoid excess in Cushing's syndrome contributes to complications such as increased visceral adipose tissue, obesity, impaired glucose tolerance, diabetes mellitus, hypertension, hyperlipidemia, osteoporosis, and neuropsychiatric comorbidities, and is associated with a significantly increased mortality [35–38]. Type II diabetes is a well-known cause of heart failure [39]. In our study, except for Cushing's syndrome, we also found the Glucagon signaling pathway in DE lncRNAs-enriched pathways and Type II diabetes mellitus in DE miRNA-enriched pathways, suggesting HYJJ might have regulation in CHF induced by Cushing's syndrome-related Type II diabetes.

Neurotransmitter noradrenaline released by the sympathetic neurons increases heart rate by activating *β*-adrenergic receptors on cardiomyocytes [40]. In the heart, increases in the inotropic, chronotropic, and lusitropic states are primarily brought about by the stimulation of *β*-adrenergic receptors [41]. Activation of *β*-AR leads to large increases in the generation of arrhythmogenic spontaneous Ca^2+^ waves (SCaWs), especially in cells from heart failure animal models [42]. It has been reported that the cAMP/PKA signaling was initiated by *β*-adrenergic receptors 3 [43, 44]. In our study, DE lncRNAs and DE miRNA shared common signaling pathways-Adrenergic signaling in cardiomyocytes. The pathways, including retrograde endocannabinoid signaling, dopaminergic synapse, calcium signaling pathway, and serotonergic synapse, were enriched with DE lncRNAs, while the pathways, including neuroactive ligand-receptor interaction, cortisol synthesis and secretion, cAMP signaling pathway, retrograde endocannabinoid signaling, and cardiac muscle contraction, were enriched with DE miRNAs. Those data suggested that HYJJ provided cardioprotective effects of CHF through adrenergic signaling in cardiomyocytes to regulate the calcium signaling pathway, and the effects might be related to neuromodulation and the cAMP signaling pathway.

In addition, we also observed hypertrophic cardiomyopathy (HCM) and arrhythmogenic right ventricular cardiomyopathy (ARVC) were enriched with DE miRNA. Cardiomyopathy is the leading cause of heart failure and mainly consists of the following four types: DCM, HCM, restrictive cardiomyopathy, and ARVC [45], so we speculated that HYJJ could be of potential benefit in the treatment of HCM and ARVC.

MAPK signaling pathway was observed to enrich with DE miRNA. MAPK pathway regulation and controlled expression of MAPK target genes are essential in mediating diverse physiologic outcomes including cellular transformation, tumorigenesis, developmental disorders, cardiac hypertrophy, and heart failure [46]. Moreover, the MAPK signaling pathway, such as ERK1/2, JNK, and p38MAPK, are demonstrated to be involved in that heart disease pathogenesis [47–50]. Our study suggested that HYJJ might regulate the expression of the MAPK signaling pathway to improve CHF.

Finally, one lncRNA-miRNA interaction of MSTRG.598.1 and Lilrb2 was observed in the HY vs. mod comparison. Lilrb2 is leukocyte immunoglobulin-like receptor B2. A study showed that high expression of Lilrb2 was identified in patients with myocardial infarction [51, 52], and downregulated Lilrb2 could visibly improve cardiomyocyte injury by regulating cell activity and apoptosis [52]. In our study, increased MSTRG.598.1 level downregulated Lilrb2 expression in the HY group and Lilrb2 miRNA was enriched in the development pathway, suggesting HYJJ might improve CHF via inhibiting MSTRG.598.1/Lilrb2 pathway. However, there is still no evidence that MSTRG.598.1 targeting acts on Lilrb2. Hence, more in-depth investigations are required in the future.

There are a few limitations of the present study. First of all, the sample size is limited which does not find more differences; therefore, more samples will be needed in the future. Notably, this was a pilot study and results must be considered preliminary but the initial findings of the study would be helpful to the follow-up study confirmation. As a next step, our group will conduct a further study to validate those molecular mechanisms.

## 5. Conclusion

The present study showed the cardioprotective effects of HYJJ in CHF via reducing myocardial apoptosis. Transcriptome results revealed that HYJJ might contribute to improving CHF via pathways, including Adrenergic signaling in cardiomyocytes, neuromodulation signaling pathway, cAMP signaling pathway, MAPK signaling pathway, and MSTRG.598.1/Lilrb2 pathway. Further studies would contribute to a better understanding of the cardioprotective roles played by HYJJ in the hypertrophied arena.

## Data Availability

The initial data used to support the findings of this study are available from the corresponding author upon request.

## Supplementary Materials

Supplementary Materials Table S1 shows ELISA detecting the contents of LVIDd, LVIDs, EF, FS, cTn-I, and NT-proBNP in rat serum. Table S2 shows the expression level of Western blot assay c-caspase 3 in myocardial tissue.
