# Supplementary material for: Analysis of Function Role and Long Noncoding RNA Expression in Chronic Heart Failure Rats Treated with Hui Yang Jiu Ji Decoction
Source: J Healthc Eng. 2023 Jan 17;2023:7438567. doi: 10.1155/2023/7438567 (PMC9873466; doi:10.1155/2023/7438567)
Supplement: Supplementary Materials — Table S1 shows ELISA detecting the contents of LVIDd, LVIDs, EF, FS, cTn-I, and NT-proBNP in rat serum. Table S2 shows the expression level of Western blot assay c-caspase 3 in myocardial tissue. [file 7438567.f1.docx]

Table S1 The table for figure1 statistical results

|  | LVIDd (mm) | LVIDs (mm) | EF (%) | FS (%) | cTn-Ⅰ (ng/mL) | NT-proBNP (pg/mL) |
| --- | --- | --- | --- | --- | --- | --- |
| control | 584.33±25.55 | 385.00±42.02 | 80.67±6.28 | 47.33±3.50 | 1.194±0.140 | 95.153±4.063 |
| CHF | 813.67±34.75 | 614.67±42.47 | 42.00±3.41 | 22.00±3.35 | 1.448±0.055 | 125.581±5.76 |
| HYJJ | 636.00±15.26 | 425.00±54.26 | 58.00±6.03 | 40.00±4.29 | 1.209±0.118 | 107.383±9.66 |
| Digoxin | 620.67±39.28 | 420.67±39.22 | 57.17±5.64 | 40.17±3.13 | 1.233±0.063 | 107.441±7.706 |
| F | 69.10 | 32.11 | 51.12 | 54.27 | 8.33 | 18.65 |
| P | 0.000 | 0.000 | 0.000 | 0.000 | 0.001 | 0.000 |

Table S2 The table for figure2 statistical results

|  | control | CHF | HYJJ | Digoxin | F | P |
| --- | --- | --- | --- | --- | --- | --- |
| Apoptosis rate (%) | 0.03±0.01 | 12.69±0.98 | 4.81±0.66 | 4.54±0.35 | 437.70 | 0.000 |
| Mean fluorescence intensity of c-caspase3 | 25.076±1.848 | 34.839±3.863 | 30.269±1.154 | 29.576±3.462 | 12.12 | 0.000 |
| Relative protein level of c-caspase3 | 1.00±0.10 | 1.63±0.11 | 1.32±0.07 | 1.24±0.08 | 23.80 | 0.000 |
